# The transcription factor Spalt and human homologue SALL4 induce cell invasion via the dMyc-JNK pathway in *Drosophila*

**DOI:** 10.1242/bio.048850

**Published:** 2020-03-24

**Authors:** Jie Sun, Junzheng Zhang, Dan Wang, Jie Shen

**Affiliations:** Department of Entomology and MOA Key Laboratory for Monitory and Green Control of Crop Pest, China Agricultural University, Beijing 100193, China

**Keywords:** Spalt, SALL4, JNK pathway, dMyc, Cell invasion

## Abstract

Cancer cell metastasis is a leading cause of mortality in cancer patients. Therefore, revealing the molecular mechanism of cancer cell invasion is of great significance for the treatment of cancer. In human patients, the hyperactivity of transcription factor Spalt-like 4 (SALL4) is sufficient to induce malignant tumorigenesis and metastasis. Here, we found that when ectopically expressing the *Drosophila* homologue *spalt* (*sal*) or human *SALL4* in *Drosophila*, epithelial cells delaminated basally with penetration of the basal lamina and degradation of the extracellular matrix, which are essential properties of cell invasion. Further assay found that *sal*/*SALL4* promoted cell invasion via dMyc-JNK signaling. Inhibition of the c-Jun N-terminal kinase (JNK) signaling pathway through suppressing *matrix metalloprotease 1*, or *basket* can achieve suppression of cell invasion. Moreover, expression of *dMyc*, a suppressor of JNK signaling, dramatically blocked cell invasion induced by *sal*/*SALL4* in the wing disc. These findings reveal a conserved role of *sal*/*SALL4* in invasive cell movement and link the crucial mediator of tumor invasion, the JNK pathway, to SALL4-mediated cancer progression.

This article has an associated First Person interview with the first author of the paper.

## Abstract (precis)

**Summary:** We investigated the molecular mechanism of how sal/SALL4 regulates cell invasion and found that this process depends on the dMyc-JNK pathway.

## INTRODUCTION

Spalt-like (Sall) gene family proteins are zinc finger transcription factors evolutionarily conserved in many organisms from *Caenorhabditis* *elegans* to human beings. These proteins can act as both transcriptional repressors and activators in different contexts (de Celis and Barrio, 2009; Sánchez et al., 2011). They play instrumental roles in stem cell development, cell specification and morphogenesis, cancer progression and inherited disorders (Sweetman and Münsterberg, 2006; de Celis and Barrio, 2009). Understanding the regulation of *Sall* genes is vital to decipher their biological functions.

The first member of the *Sall* gene family, *spalt* (*sal*), was identified as a homeotic gene during *Drosophila* embryonic development (Frei et al., 1988; Kühnlein et al., 1994). There are two *Drosophila spalt* homologues, *spalt major* (*salm*) and *spalt-related* (*salr*), which have complementary functions (Barrio et al., 1996, 1999). Numerous studies have been devoted to the role of *sal* in patterning and growth control of the *Drosophila* wing imaginal disc, an epithelial tissue that proliferates during larval development. In the wing disc, the expression of *sal* is activated by Decapentaplegic (Dpp) signaling in specific regions and leads to tissue growth (de Celis et al., 1996; Barrio and de Celis, 2004; Doumpas et al., 2013; Akiyama and Gibson, 2015). Loss of *sal* shows abnormal vein formation and reduction in wing size (de Celis et al., 1996; Grieder et al., 2009; Wang et al., 2017). At the cellular level, mitotic cells are strongly reduced in *sal* mutant wing discs (Organista and De Celis, 2013). Cell death pathways and the JNK signaling are activated in *sal* knockdown cells, but these two processes only have a minor role in generating the *sal* mutant phenotypes (Organista and De Celis, 2013; Organista et al., 2015). Conversely, ectopic *sal* expression promotes cell proliferation (Skottheim Honn et al., 2016; Wang et al., 2017) via positive regulation of the microRNA *bantam* (Wang et al., 2017). These results suggest that *sal* is vital in organ size control by accelerating cell proliferation, but the relation of *Drosophila sal* to tumorigenesis is not yet known.

In vertebrates, there are four *Sall* paralogues, named *Sall1* to *Sall4*. All four vertebrate Sall members are involved in embryonic development and their mutations lead to severe genetic disorders (Sweetman and Münsterberg, 2006; de Celis and Barrio, 2009). Particularly, *SALL4*, a mutation that causes Okihiro syndrome (Al-Baradie et al., 2002; Kohlhase et al., 2002), is highly expressed during embryonic development and plays a crucial role in maintaining pluripotency and self-renewal of embryonic stem cells (Wu et al., 2006; Zhang et al., 2006; Yang et al., 2008a). As tissues and organs mature, the expression of *SALL4* is gradually decreased. By contrast, there is substantial evidence that *SALL4* is highly upregulated in numerous human cancers and regulates multiple cellular processes responsible for cancer progression (Zhang et al., 2015). First, *SALL4* regulates the self-renewal of cancer stem cells by targeting a variety of genes, such as upregulation of *Bmi-1*, *Wnt*/*β-catenin* and *HoxA9* and repression of *PTEN*, a tumor suppressor gene (Ma et al., 2006; Lu et al., 2009; Li et al., 2013; Zhang et al., 2014). Second, *SALL4* regulates cell proliferation and apoptosis. Overexpressing *SALL4* in liver cancer cell lines enhances cell proliferation through *Cyclin D* expression (Oikawa et al., 2013). In addition, SALL4 negatively regulates the transcription of apoptotic genes (Yang et al., 2008b; Li et al., 2015) through activating the oncogene *Bmi-1* (Yang et al., 2007; Lu et al., 2011). Correspondingly, silencing of *SALL4* results in less proliferation and differentiation (Elling et al., 2006; Sakaki-Yumoto et al., 2006; Zhang et al., 2006), which is significantly correlated with cell cycle arrest (Böhm et al., 2007; Lu et al., 2011; Oikawa et al., 2013; Zhang et al., 2017) and/or increased apoptosis (Li et al., 2015; Zhang et al., 2017). Third, *SALL4* regulates cell migration and invasion. *SALL4* improves epithelial-mesenchymal transition (EMT), as indicated by increasing Twist1 and N-cad expression and decreasing expression of E-cad (Zhang et al., 2014; Li et al., 2015; Liu et al., 2015). The EMT activator ZEB1 (Itou et al., 2013) and oncogene *cMyc* (Yang et al., 2008a; Li et al., 2015; Liu et al., 2015) are positively regulated by *SALL4*, therefore leads to EMT. Transplantation of *SALL4*-expressing cells into immunodeficient mice gives rise to subcutaneous tumor growth and tumefaction of many organs (Ma et al., 2006; Oikawa et al., 2013). Lastly, *SALL4* is associated with drug resistance, which, in turn, hampers treatment of tumor cell growth (Oikawa et al., 2013; Liu et al., 2015). Thus, *SALL4* plays an essential role in regulating tumorigenesis, tumor growth and tumor progression. Yet, how *SALL4* regulates invasive cell movement at the molecular level needs to be elucidated.

In this article, we make use of a *Drosophila* genetic model for epithelial tumor invasion to explore the molecular mechanism of *SALL4* in cancer cell invasion and metastasis. Overexpressing the *Drosophila sal* or human *SALL4* generated migrating cells with invasive behavior in the *Drosophila* larval tissues. The additional cellular and genetic data revealed that *sal*/*SALL4-*induced cell invasion depended on dMyc-JNK signaling and was independent of the apoptosis pathway. These results provide new insights into the molecular mechanisms of *sal*/*SALL4*-induced cancer invasion and metastasis.

## RESULTS

### *sal*/*SALL4* hyperactivation stimulates cell invasion

**Fig. 1.**
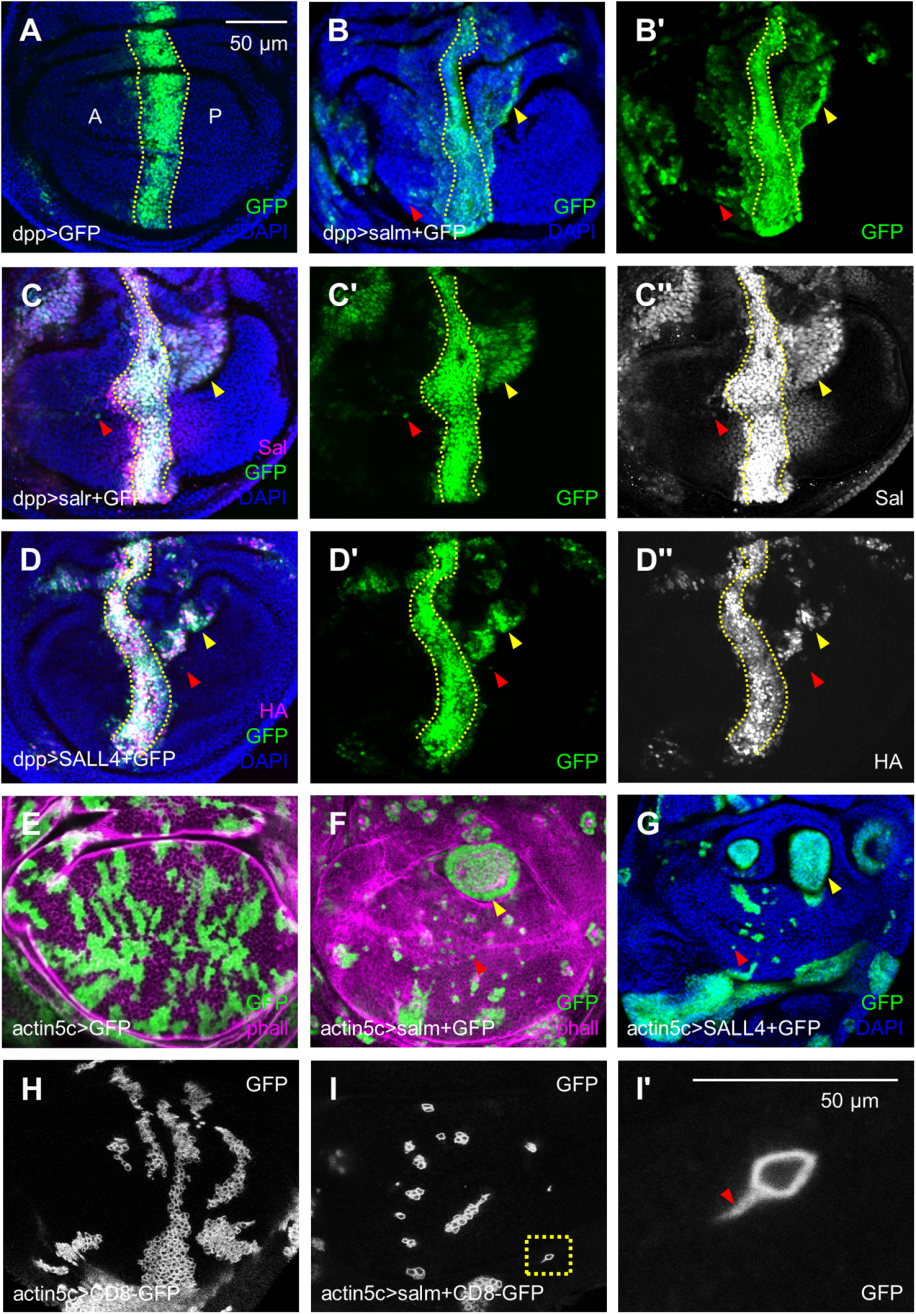
***sal/SALL4* induces cell invasion in** **the larval body and wing disc.** (A) GFP signal driven by the *dpp-Gal4* was expressed in a stripe in the anterior wing disc. A indicates the anterior compartment and P is posterior compartment. Dashed lines in A-D contour the rough *dpp-Gal4* region. In this and subsequent figures, wing imaginal discs were oriented anterior left and dorsal up. The developmental stages were late third-instar and the x-y images were focused on the middle section of the wing pouch and hinge region, unless indicated elsewhere. (B–D) Cells expressing *salm* (B), *salr* (C), or *SALL4* (D) in the *dpp-Gal4* domain invaded into both A and P compartments. In most cases, there was a groove in the pouch region due to *sal* discontinuity regulated cell sorting. The red arrowheads indicate the single migrating cells and the yellow arrowheads indicate the cell mass in B–G. (E–G) GFP-labeled clone cells. Compared with the control (E), cells overexpressing *salm* (F) or *SALL4* (G) tended to disperse into the single cell level (red arrowheads). The yellow arrowheads represent the hyperproliferative tumor cells. (H) Control clones that expressing the membrane CD8-GFP. (I) The filopodia-like structure appeared in the moving cells shown by CD8-GFP. I′ was the magnification of the box in I. The arrowhead shows the membrane protrusion. Scale bars: 50 µm.

Given the expression level of *SALL4* is increased in many types of tumors, to uncover whether *SALL4* is capable of inducing cell migration and invasion *in vivo*, we increased Sal levels in a central region within the *spalt* expression domain by expressing *salm*, *salr* or human *SALL4*. In the wing disc, when GFP was expressed in the *dpp-Gal4* domain in the wild-type background, the boundary (indicated by dotted lines in Fig. 1A) was relatively linear and no GFP-positive cells could be found in the P compartment. In contrast, a significant number of GFP-labeled cells were present both in anterior and posterior regions far away from the *dpp-Gal4* domain when *sal*/*SALL4* was overexpressed (Fig. 1B–D). These cells were largely two types. One was grouped cells extruding into the posterior region, which had connections to the major *dpp* expression region (Fig. 1B–D, yellow arrowheads) and may be either proliferated (Wang et al., 2017) or migrated from the main part. The other was single cells, which were separated from the *dpp* expression region (Fig. 1B–D, red arrowheads) and probably migrated from the main part. Because the *dpp* region is anterior cell fate, if these anterior GFP cells emerge in the posterior region, it means they could go across the compartment boundary and invade into the posterior region (Fig. S1). Hence, we considered the GFP signals in the P compartment of the pouch region as invasive cells. To verify that the GFP-tagged cells represent the *sal*/*SALL4*-overexpressing cells, Sal and SALL4 were labeled with anti-Sal and anti-HA tag antibodies, respectively. Cell migration occurred exactly in the Sal/HA positive regions (Fig. 1C″,D″). These data demonstrate that *Drosophila salm*, *salr* and human *SALL4* are highly conserved. For convenient genetic manipulation, we used human SALL4 and one of the *Drosophila* homologues (either *salm* or *salr*) for the following experiments.

Next, clones were performed to further confirm that *sal*/*SALL4* regulates cell movement. In control clones, cells descending from one progenitor tended to remain clustered and the rugged clone outlines (GFP positive cells) showed similar adhesive properties with their unmarked neighbors (GFP negative cells) (Fig. 1E). When *sal*/*SALL4* was overexpressed, some clone cells were dispersed to the single cell level (Fig. 1F,G, red arrowheads), which is similar to expressing another Dpp target gene *optomotor-blind* (Shen et al., 2014), indicating increased mobility of *sal*/*SALL4*-expressing cells. Tumor-like proliferating cell clusters were seen in the hinge region (Fig. 1F,G, yellow arrowheads), a tumor hotspot where tumors often originate (Tamori et al., 2016). Co-expression of the membrane marker CD8-GFP with *sal* showed that the migrating cells had filopodia-like structures (Fig. 1I), which is a property of migratory and invasive cells (Shen et al., 2014). Taken together, our results demonstrate that the *Drosophila salm*, *salr* and human *SALL4* are highly conserved in stimulating cell proliferation and cell motility in the wing disc.

To examine whether *sal*/*SALL4* is able to modulate cell movement in other tissues, we turned to the salivary gland, where *sal* was endogenously expressed at a moderate level (Fig. S2A). Overexpressing *sal*/*SALL4* by *AB1-Gal4* triggered cell invasion throughout the body (Fig. S2C,D). After dissecting the body wall of third-instar larvae, invading cells (GFP positive) were detected and completely co-localized with the HA antibody staining (Fig. S2E′), confirming that the GFP-labeled invading cells showed high *sal*/*SALL4* expression. Collectively, our data suggest that ectopic *sal/SALL4* expression is sufficient to trigger cell invasion into other tissues.

### *sal*/*SALL4*-hyperactive cells give rise to disruption of cell polarity

**Fig. 2.**
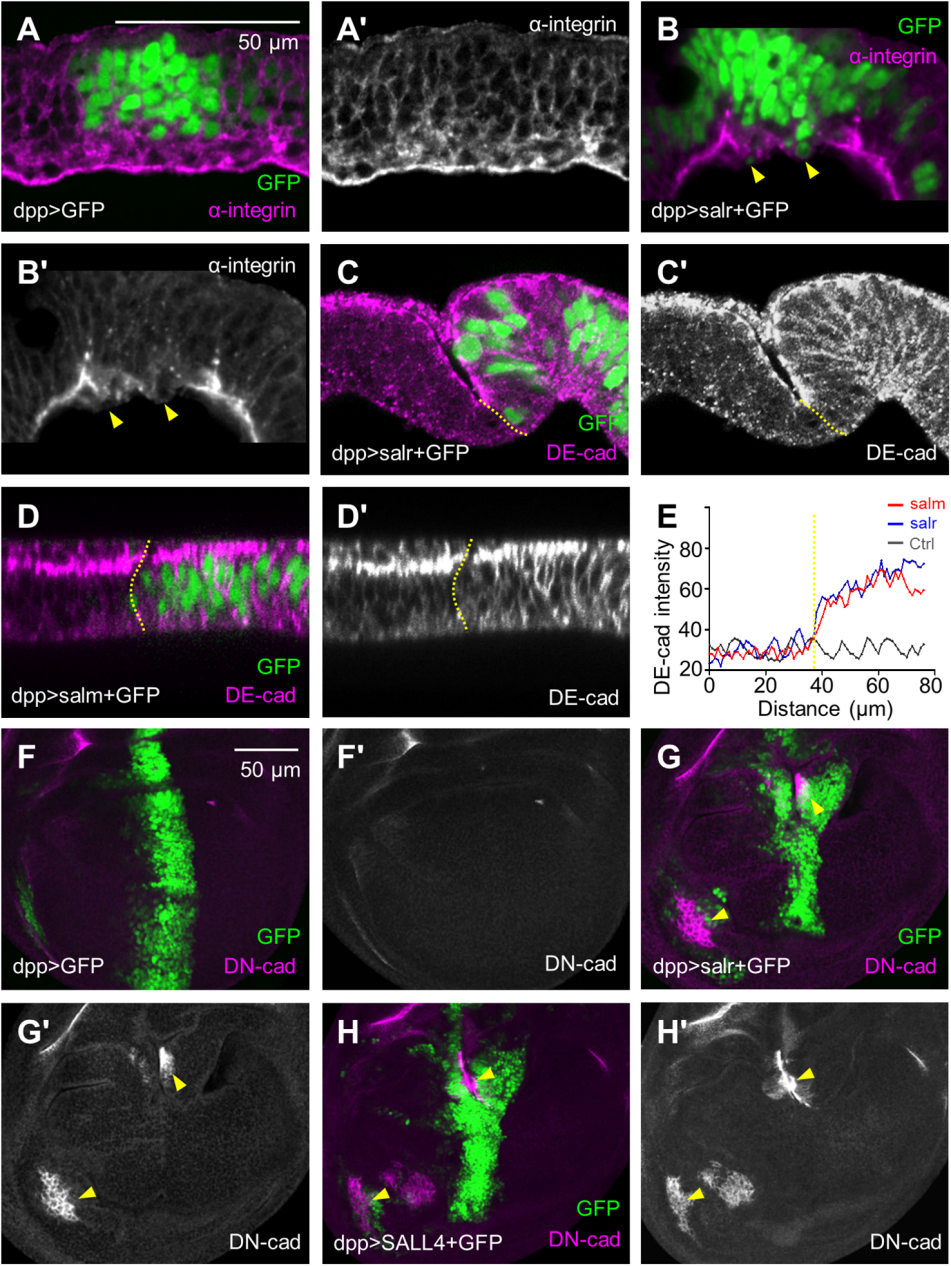
**The apico-basal polarity is disrupted in *sal/SALL4*-overexpressing wing discs.** (A) α-integrin was specifically concentrated at the basement membrane. Wing discs as shown in Fig. 1 were sectioned along the x-z axis and images here showed the side view. In all x-z scans apical cells were up and anterior cells were left. (B) Expressing *salr* induced cell extrusion and ECM degradation. Arrowheads show the degradation of integrin in extrusion cells. (C) DE-cad was rearranged in cells overexpressing *salr*. The apical DE-cad was comparable in *salr*-overexpressing and non-overexpressing cells, but the lateral localization was increased in *salr*-overexpressing cells (GFP expressing regions). Dashed lines in C-E mark the boundary of GFP-expressing and non-expressing cells. (D) The lateral DE-cad was increased in cells overexpressing *salm*. (E) The profile of DE-cad fluorescence intensity. (F–H) The EMT marker DN-cad occurred in *salr/SALL4*-overexpressing cells. Arrowheads indicate the ectopic DN-cad. Scale bars: 50 µm.

The invasive behavior of transformed cells is commonly associated with EMT, whose characteristics include increased cell motility, destabilization of adhesion junctions and loss of cell polarity. In order to better visualize the property of *sal/SALL4*-overexpressing cells, we performed cryosectioning in the wing discs*.* At the late third-instar stage, the basal membrane of wing disc epithelia was marked by α-integrin (Fig. 2A). In contrast, the *salr*-overexpressing cells, which were extruded toward the basal side of epithelia, were deficient in α-integrin expression and substantially lost contact with the epithelia (Fig. 2B, arrowheads). These observations suggest that the *salr*-hyperactive cells were penetrating the extracellular matrix (ECM) during invasive migration. The apical DE-cadherin (DE-cad) protein level did not change significantly, but its localization in cytoplasm and basal distribution were increased (Fig. 2C–E). Cytoplasmic distribution of soluble E-cad, which is generated from extracellular cleavage by matrix metalloprotease (Mmp), is known to promote epithelial cell extrusion (Grieve and Rabouille, 2014). Interestingly, hyperactivation of *salr/SALL4* resulted in upregulation of the mesenchymal fate marker DN-cadherin (DN-cad) (Fig. 2G,H), indicating that *sal/SALL4* overexpression induces some consequence related to EMT.

As the large size of salivary gland cells makes it easier to observe the cell morphology and cellular protein localization, we used this tissue to further observe the changes of cell polarity. The apical markers DE-cad and β-catenin/Armadillo (Arm), which were expressed on the cell membrane (Fig. S3A,C), were both mis-localized cytoplasmically in *sal*-expressing cells (Fig. S3B,D). We further marked the apical membrane by antibody against Discs large (Dlg). Dlg was apparently disorganized in *sal*-expressing cells (Fig. S3F). A severe disruption of the actin and microtubule cytoskeleton may have contributed to the disruption of apical polarity due to the morphological changes of *sal*-expressing cells (Fig. S3H) (Tang et al., 2016). The above data suggest that *sal* activation promotes cell invasion by disruption of the apico-basal polarity.

### JNK signaling is essential for *sal*/*SALL4* activation-induced cell invasion

**Fig. 3.**
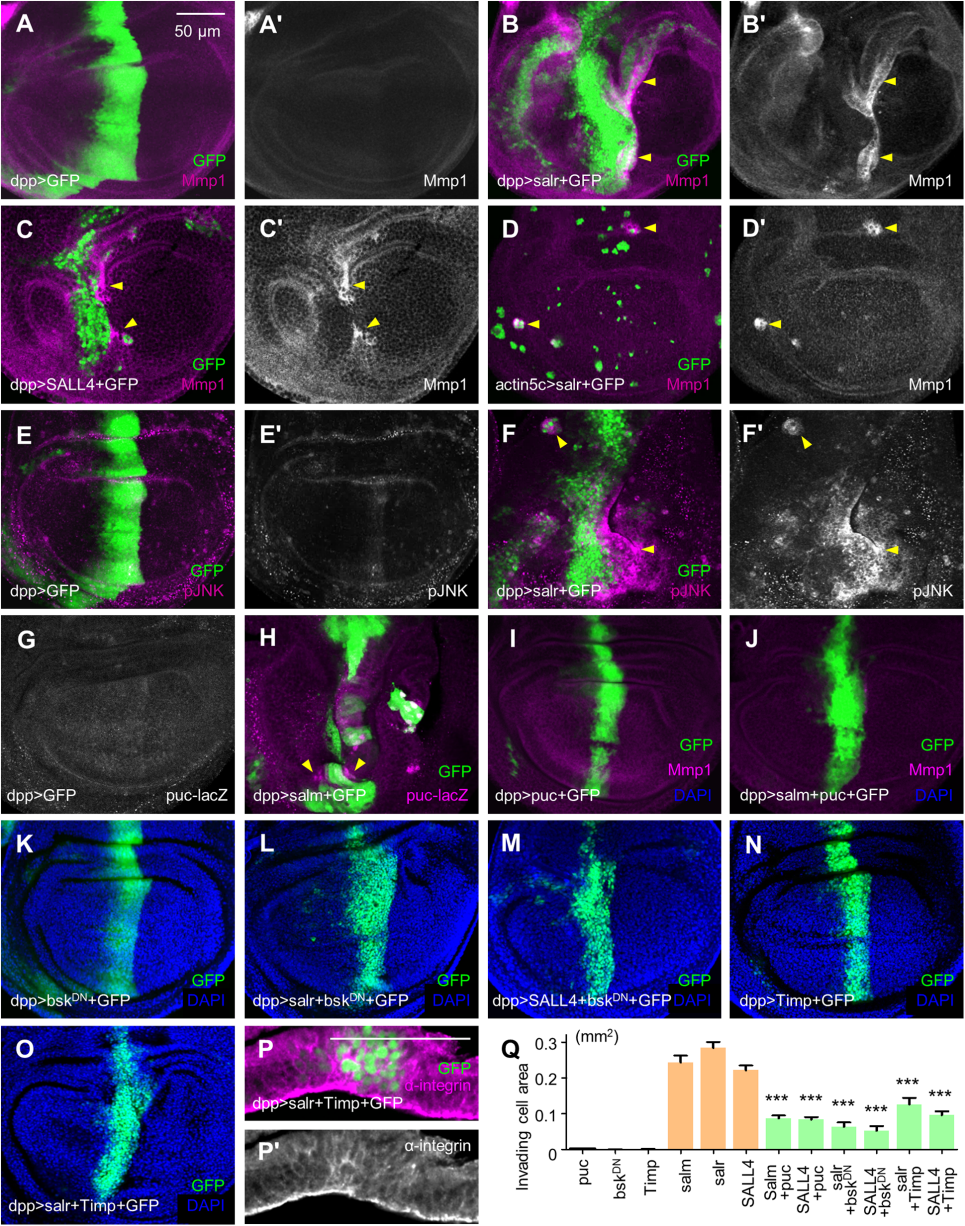
***sal/SALL4* promotes cell invasion through the JNK signaling.** (A) Wild-type cells had no obvious JNK activation as indicated by the Mmp1 staining. (B,C) The Mmp1 level was upregulated in *salr*/*SALL4*-overexpressing wing discs. Arrowheads in B-H indicate the increased JNK signaling. (D) Mmp1 was activated in clone cells overexpressing *salr*. (E) pJNK expression was slightly activated in the central stripe of wild-type wing discs. (F) Overexpression of *salr* promoted JNK phosphorylation. (G) *puc* was not activated in the control wing disc. (H) *puc* was activated in the *salm-*overexpressing cells. Arrowheads show the autonomously increased JNK signaling and non-autonomous increase in the surrounding cells. (I,J) Co-expression of *salm* and *puc* suppressed *salm*-induced cell invasion as well as the Mmp1 level. (K–M) Cell invasion induced by *salr*/*SALL4* was significantly inhibited by *bsk^DN^*. (N,O) Co-expression of *salr* and *Timp* suppressed *salr*-induced cell invasion. (P) Co-expression of *salr* and *Timp* suppressed *salr*-induced cell extrusion. (Q) Quantification of the area of invading cells into the P compartment. Each genotype was quantified for 30 wing discs. *** represents *P*<0.001 (two-tailed one-way ANOVA tests for each genetic interaction with *salm*, *salr* and *SALL4* overexpression). Error bars indicate s.e.m. Scale bars are the same except in P. Scale bars: 50 µm.

Because the JNK pathway is an essential pathway driving tumor growth and invasion, we investigated whether the JNK pathway mediates *sal*/*SALL4* overexpression-induced cell invasion. Degradation of the ECM components and basement membrane requires the activity of Mmp1, a transcriptional target of JNK signaling (Uhlirova and Bohmann, 2006). We first examined the Mmp1 level. *salr*/*SALL4* overexpression by *dpp-Gal4* or in clone cells within the wing discs led to a strong increase in Mmp1 protein level (Fig. 3B–D). The deposition of Mmp1 was also found in the salivary gland (Fig. S4B, dotted lines). Then, the JNK signaling level was probed by a specific antibody against the activated JNK isoform pJNK. The pJNK level was elevated when *salr* was overexpressed (Fig. 3F). The JNK pathway target *puckered* (*puc*) was transcriptionally upregulated (Fig. 3H). Besides in the *sal*/*SALL4*-expressing regions, the location of Mmp1, pJNK and *puc* usually occurred at or close to the edge of *salr*/*SALL4*-overexpressing domains (arrowheads in Fig. 3). The non-autonomous activation of JNK pathway in neighboring wild-type cells may also contribute to invasive cell migration, such as in mutant clones for the tumor-suppressor *scrib* (Ohsawa et al., 2011).

To examine whether JNK is required for *sal*/*SALL4-*induced cell invasion, we blocked JNK signaling by expressing several JNK pathway inhibitors. As *puc* is a JNK-specific inhibitor (Martin-Blanco et al., 1998), increasing *puc* expression is thought to inhibit the JNK activity. As a result, the invasive migration in *sal/SALL4-*overexpressing wing discs was repressed by expressing *puc* (Fig. 3I,J). The Mmp1 level, both in *sal*/*SALL4*-expressing regions and adjacent wild-type cells, was rescued (Fig. 3I,J), indicating that the non-autonomous activation of JNK pathway depends on JNK signals from the *sal*/*SALL4*-expressing cells. A dominant-negative form of the *Drosophila JNK* homologue *basket* (*bsk^DN^*) also greatly repressed *salr*/*SALL4*-induced cell invasion (Fig. 3L,M). Consistently, downregulation of *Mmp1* by expressing *tissue inhibitor of matrix metalloprotease* (*Timp*) (Visse and Nagase, 2003) compromised *salr*-induced cell invasion (Fig. 3O). In cryosectioning discs, the restoration of basal membrane integrity by *Timp* was apparent (as indicated by anti-α-integrin staining, Fig. 3P). Statistically, the GFP area in the P compartment was significantly reduced when JNK signaling was repressed. The area of invading cells was reduced more than 60% compared with that of *salm*, *salr*, or *SALL4* (Fig. 3Q). The above data suggest that inhibition of the JNK pathway largely reduces *sal*/*SALL4*-induced cell invasion and epithelial disruption.

As the activation of JNK signaling is often accompanied by the appearance of apoptosis and apoptosis can cause delamination and/or migration of epithelial cells (Rudrapatna et al., 2013; Gorelick-Ashkenazi et al., 2018), we assessed the function of apoptosis in *sal*/*SALL4*-overexpressing cells. Caspase-3 (Cas3) was activated in and close to the *salr*/*SALL4*-overexpressing domain (Fig. S5B,C, yellow arrowheads), as well as non-autonomously activated elsewhere (Fig. S5B,C, red arrowheads). Further TUNEL assay showed that the migrating cells were not dead cells (Fig. S5D,E). When apoptosis was inhibited by overexpression of *p35*, an inhibitor of the caspase drICE, *salr*/*SALL4*-expressing cells still maintained the ability of horizontal invasion (Fig. S5G,H). To avoid the fact that expressing *p35* induces ‘undead’ cells to produce migration signals (Martin et al., 2009), we used *Diap1* (Fan and Bergmann, 2008) to suppress caspase Dronc-mediated cell death. Co-expression of *Diap1* and *salr*/*SALL4* still induced a large number of invading cells (Fig. S5J,K). Thus, co-expression of *p35*/*Diap1* and *salr*/*SALL4* cannot rescue *sal*/*SALL4*-induced cell invasion. Apoptosis does not play a major role in this process.

### dMyc is repressed by *sal*/*SALL4*

**Fig. 4.**
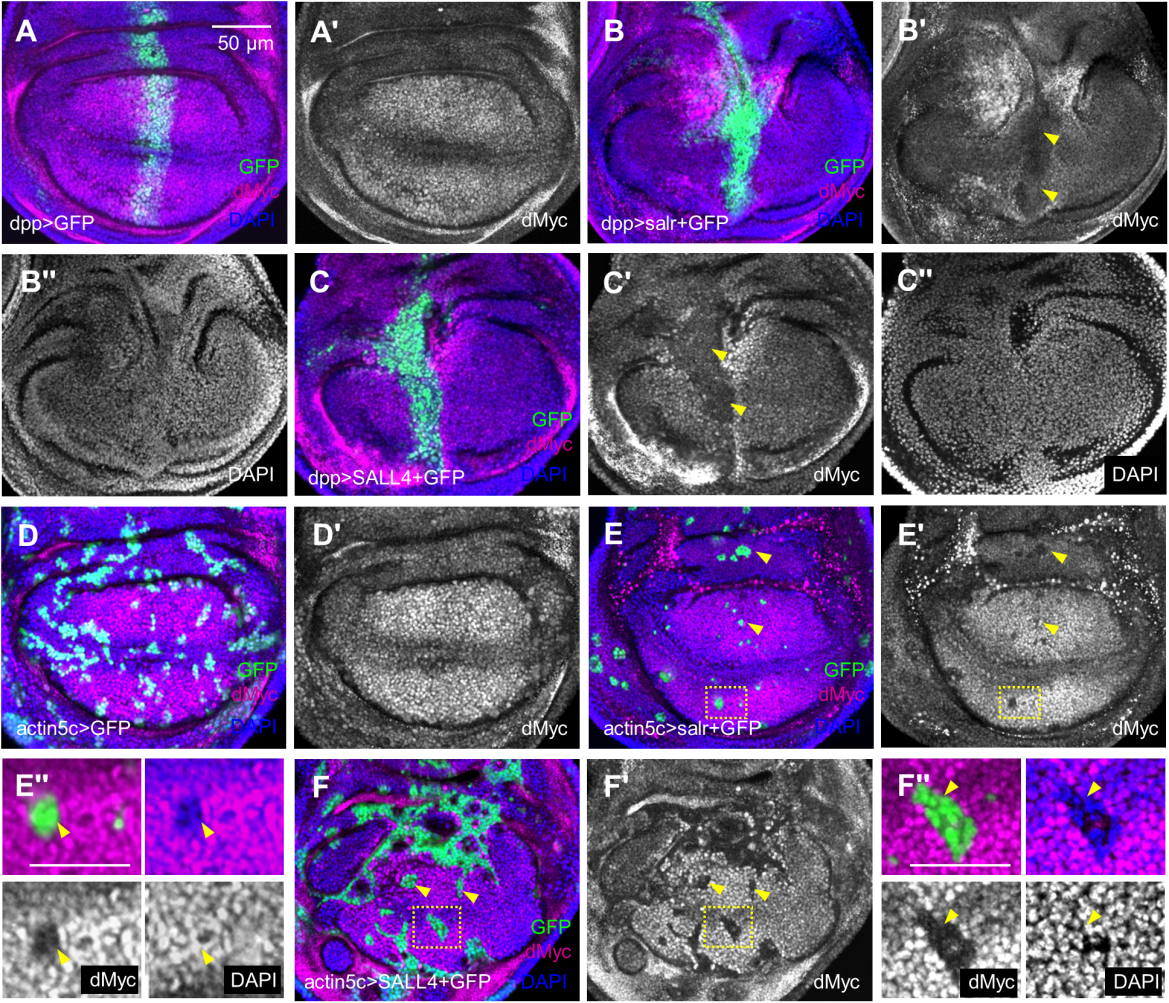
***sal/SALL4* inhibits dMyc expression.** (A) dMyc was expressed in the wing discs. (B,C) dMyc was downregulated in *salr/SALL4*-overexpressing cells. Arrowheads in B′ and C′ indicate the areas that dMyc was obviously repressed. (D–F) dMyc was reduced in *salr/SALL4*-overexpressing clone cells. The arrowheads mark the clone cells. E″ and F″ are higher resolution images for box areas in E and F. Scale bars: 50 µm except in the higher resolution images where scale bars are 25 µm.

The human *MYC* is an oncogene that contributes to tumorigenesis and metastasis. So does the single *Drosophila* homologue *dMyc* (Dang, 2012). Previous reports also showed that loss of *dMyc* promotes cell migration by activating JNK signaling (Ma et al., 2017a; Tavares et al., 2017). Here, overexpression of *salr*/*SALL4* led to a downregulation of the dMyc level in the *dpp-Gal4* domain (Fig. 4B′,C′, arrowheads). To confirm the regulation by *sal*/*SALL4*, we produced *salr*/*SALL4*-overexpressing clones in which the dMyc level was consistently downregulated (Fig. 4E,F, arrowheads). Higher-resolution images illustrated that dMyc was reduced in clone cells (Fig. 4E″,F″, arrowheads). Consistently, dMyc was reduced in the salivary gland (Fig. S4D, dotted lines). Therefore, dMyc was cell-autonomously repressed by *sal*/*SALL4*.

### *dMyc* suppresses cell invasion induced by *sal*/*SALL4* overexpression

**Fig. 5.**
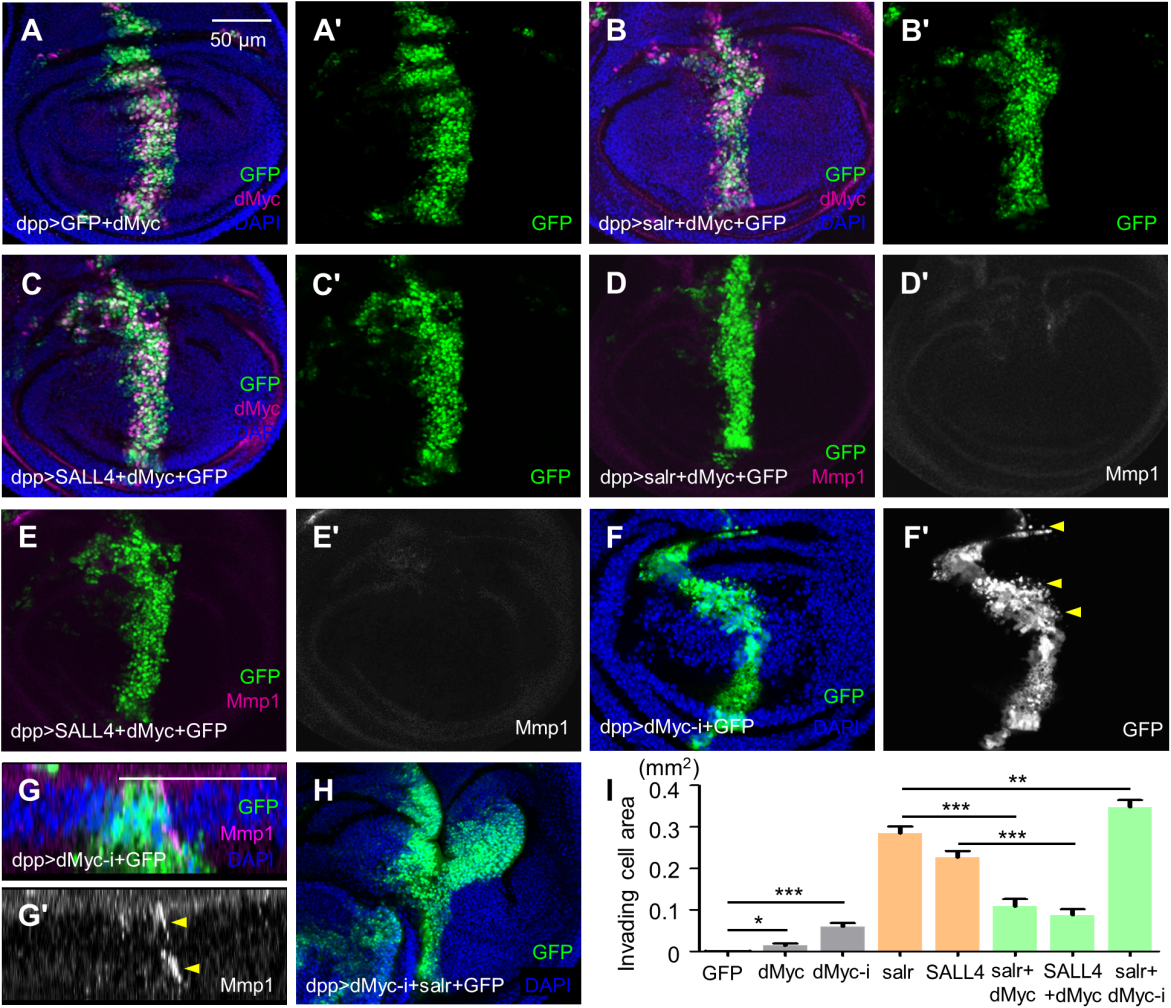
***sal/SALL4*-induced cell invasion depends on dMyc expression.** (A) Expressing *dMyc* showed subtle migration phenotype. The outline of GFP at the A/P compartment boundary was not as smooth as that in previous *dpp>GFP* controls. (B,C) Overexpression of *dMyc* greatly repressed *salr/SALL4*-induced cell invasion. (D) Mmp1 was not activated in the wing discs co-expressing *salr* and *dMyc*. (E) Mmp1 level was not increased in the wing discs co-expressing *SALL4* and *dMyc*. (F) Downregulation of *dMyc* alone induces cell migration. Arrowheads indicate single cell migration into the P compartment. (G) The Mmp1 level was upregulated in *dMyc*-knockdown wing discs. Arrowheads show the high Mmp1 expression in the *dMyc*-knockdown cells. (H) Co-expression of *salr* and *dMyc-RNAi* (*dMyc-i*) exacerbated *salr*-induced cell invasion. (I) Quantification of invading cell areas. Each genotype was quantified for 30 wing discs. *** represents *P*<0.001 (two-tailed pairwise comparison of *t*-tests). Error bars indicate s.e.m. Scale bars are the same except in G. Scale bars: 50 µm.

Although overexpression of dMyc showed weak cell migration in the wing disc (Fig. 5A), we attempted to rescue *sal*/*SALL4*-induced cell invasion by expressing *dMyc*. Co-expression of *dMyc* and *salr*/*SALL4* significantly reduced the cell invasion rates (Fig. 5B,C). Statistical results indicate that more than 70% of the GFP cells in the P compartment was lost (Fig. 5I). At the same time, the JNK signal activated by *salr*/*SALL4* ectopic expression was repressed by *dMyc* expression as indicated by the Mmp1 staining (Fig. 5D,E). In turn, knock-down of *dMyc* by *dMyc-RNAi* showed obvious single cell movement (arrowheads in Fig. 5F′). Reducing *dMyc* also induced activation of the JNK pathway, which was more obviously seen in the x-z view (Fig. 5G). Thus, we deduce that concurrently expressing *dMyc-RNAi* and *sal*/*SALL4* will enhance *sal*/*SALL4*-induced cell invasion and the results were as expected (Fig. 5H,I). These findings demonstrate that *dMyc* inhibits the JNK signaling and the *Drosophila* epithelial cell invasion induced by *sal*/*SALL4* depends on dMyc-JNK signaling.

## DISCUSSION

Human *SALL4* has been reported to be significantly elevated in metastatic cancer cells. Here, we provide genetic evidence for a model in which *sal*/*SALL4* regulates cell invasiveness by dMyc-JNK signaling. The JNK pathway is an important cellular signaling pathway that regulates a variety of cellular activities relevant to tumorigenesis, such as cell migration, apoptosis and proliferation. JNK promotes the expression of Mmp1, which acts as an enzyme to degrade basement membrane and ECM components to promote tumor cell motility (Uhlirova and Bohmann, 2006). Manipulation of expression of many genes can lead to cell death, cell extrusion and invasive cell migration through activation of JNK signaling (Petzoldt et al., 2013; Rudrapatna et al., 2014; Ma et al., 2017a,b; Sun et al., 2019). *sal*/*SALL4* overexpression activates *Mmp1* and reducing JNK can suppress cell invasion and Mmp1 level (Fig. 3; Fig. S4). In addition to Mmp1, some other markers in the JNK pathway such as pJNK (activated *bsk*) and *puc* showed a significant increase in expression (Fig. 3). Promotion of cell invasion by *sal*/*SALL4* induction was accompanied by activation of the apoptotic pathway, but it was not dependent on apoptosis because caspase inhibition did not prevent cell invasion upon *sal*/*SALL4* expression (Fig. S5). Therefore, the JNK pathway probably mediates the role of *sal*/*SALL4* overexpression to regulate cell invasion through an apoptosis-independent mechanism.

The *MYC* gene is one of the most highly amplified oncogenes among many human cancers (Dang, 2012). For instance, in some certain cancer cells, *Myc* is upregulated through directly transcriptional activation by *SALL4* (Yang et al., 2008a; Li et al., 2015; Liu et al., 2015). Besides promoting cancer progression and metastasis, *MYC* has a bivalent role in regulating tumorigenesis and cell invasion. *MYC* restrains breast cancer cell motility and invasion through transcriptional silencing of integrin subunits (Liu et al., 2012). In *Drosophila*, *dMyc* inhibits JNK signaling in retinal progenitors to block non-autonomous glia over-migration (Tavares et al., 2017). The *Drosophila puc* gene, encoding the sole JNK-specific MAPK phosphatase and inhibitor (Martin-Blanco et al., 1998), and its mammalian homologue *Dusp10* are directly bound by Myc as shown in ChIP-sequencing data (Yang et al., 2013; Sabò et al., 2014). In *Drosophila* tissues*,* direct evidence illustrates that *dMyc* and *cMyc* activate *puc* transcription through binding to the Myc binding-motif EB3, and consequently inhibit JNK signaling to suppress cell invasion (Ma et al., 2017a). We found that *dMyc* is repressed in *sal*/*SALL4*-expressing regions and introducing *dMyc* partially rescues cell invasion (Figs 4 and 5), indicating a repressive role of *dMyc* in tumor cell migration. As Sal is a transcriptional repressor in both *Drosophila* and human cells (Sánchez et al., 2011), it is possible that Sal/SALL4 binds to *Myc* and suppresses its expression because the *cMyc* promoter has putative binding sites that are available to Zinc finger binding (Wu et al., 2015). Sall2, another emerging cancer player in the Sall family, binds to the *cMyc* promoter region and represses *cMyc* expression (Sung et al., 2012; Wu et al., 2015). Thereby, *sal*/*SALL4* may activate JNK signaling through the repression of *puc*, which is activated by d*Myc* in *Drosophila*.

Cell competition occurs when *Myc* is unevenly distributed between cells. Clones expressing high levels of *Myc* expand and eliminate the surrounding cells by apoptosis. On the contrary, downregulation of *Myc* in clones leads to their elimination (de la Cova et al., 2004; Moreno and Basler, 2004). Given *sal/SALL4*-expressing cells are relatively lower *Myc* expression, it is possible that the surrounding cells with higher *Myc* expression become competitors and eliminate those lower *Myc* expression cells. Intriguingly, *sal/SALL4*-induced migrating cells are not dead and inhibiting cell death cannot repress *sal/SALL4*-induced cell invasion (Fig. S5), so the mechanism may not be apoptosis-driven cell elimination (Levayer and Moreno, 2013; Levayer et al., 2015). Previous studies found that JNK activation in surrounding wild-type cells promotes elimination of their neighboring *scrib* mutants by activating the PVR-ELMO/Mbc-mediated engulfment pathway, and the surrounding JNK is independent of JNK activation in mutant clones (Ohsawa et al., 2011; Nagata and Igaki, 2018). Distinct from this, *sal/SALL4*-activated non-autonomous activation of JNK is dependent on JNK activation in *sal/SALL4*-expressing cells (Fig. 3J,K). Whether JNK-dependent engulfment plays a major role in *sal/SALL4*-mediated extrusion needs to be addressed in the future.

## MATERIALS AND METHODS

### *Drosophila* strains and rearing conditions

Fly lines were cultured at 25°C on standard fly food unless otherwise noted. The transgenes used were as follows: *UAS-salr* (de Celis et al., 1996), *UAS-salm* (from the Bloomington *Drosophila* Stock Center #29716, short for BL#29716), *UAS-SALL4-HA* (BL#65835), *UAS-Timp* (BL#58708), *UAS-bsk^DN^* (Weber et al., 2000), *UAS-p35* (BL#5073), *UAS-Diap1* (BL#6657), *UAS-GFP* (nuclear expression, BL#4775), *UAS-CD8-GFP* (membrane expression) (Lee and Luo, 1999), *UAS-dMyc* (BL#9674), *dMyc-RNAi* (BL#36123), *puc-lacZ* (Martin-Blanco et al., 1998), *UAS-puc* (Dobens et al., 2001), *dpp-Gal4* (Shen and Mardon, 1997), *actin5c>CD2>Gal4* (Pignoni and Zipursky, 1997), and *AB1-Gal4* (BL#1824). To promote the GFP phenotype in a larval body, *salm*, *salr*, or *SALL4*-overexpressing larvae were raised at 29°C after egg laying. Clones in the larval wing imaginal discs were generated with the genotypes *y w^1118^ hs-Flp; actin5c>CD2>Gal4 UAS-GFP/CyO; UAS-salr/UAS-SALL4-HA* by heat shock at 35.5°C for 30 min. Then, late third-instar larvae were dissected after a recovery period of 3 days at 25°C.

### Antibody staining

Dissected imaginal discs from third-instar larvae were fixed and immunostained using standard procedures for confocal microscopy. Appropriate primary antibodies and staining reagents include rhodamine-phalloidin (1:50, Invitrogen A12380, Waltham, USA), DAPI (1:500, Sigma-Aldrich 32670, Shanghai, China), rabbit anti-HA [1:500, Cell Signaling Technology (CST) #3724S, Danvers, USA], rat anti-Ci [1:200, Developmental Studies Hybridoma Bank (DSHB) 2A1, IA, USA], mouse anti-α-integrin (1:20, DSHB DK.1A4), rat anti-DE-cadherin (1:100, DSHB DCAD2), mouse anti-DN-cadherin (1:10, DSHB DN-EX #8), mouse anti-Dlg (1:10, DSHB 4F3), mouse anti-Arm (1:100, DSHB N2 7A1), mouse anti-Mmp1 (1:20, DSHB 5H7B11), rabbit anti-pJNK (1:200, CST #4668), rabbit anti-dMyc (1:400, Santa Cruz Biotechnology sc-28207, CA, USA), rabbit anti-β-galactosidase (1:2000, Promega Z378B, Madison, USA), rabbit anti-cleaved caspase-3 (1:200, CST #9661), and rabbit anti-p35 (1:500, Novus Biologicals NB100-56153, Centennial, USA). Rabbit anti-Sal antibody (1:500) was a gift from Professor Rosa Barrio at CIC bioGUNE, Spain. Secondary antibodies (1:200, Jackson ImmunoResearch, West Grove, USA) were anti-mouse Cy2 (115-225-146), Cy3 (115-165-146) and Cy5 (115-175-146); anti-rabbit Cy2 (111-225-144), Cy3 (111-165-144), and Cy5 (111-175-144); and anti-rat Cy3 (112-165-143). The samples were mounted in 50% glycerin before imaging.

### Wing disc cryosectioning

After secondary antibody staining, discs were re-fixed in freshly made 4% paraformaldehyde for 30 min and washed three times with 1× PBS, then stored in 30% sucrose solution at 4°C overnight. Wing discs were oriented in Tissue-Tek (Sakura Finetek, Japan), frozen and cut into 20 μm sections on a cryostat (YD-1900, YIDI, China). All samples were mounted in 50% glycerin before imaging.

### Imaging and statistics of invasive cell area

Imaging of prepared samples was collected by a Leica SP8 confocal microscope. Adult wing images were collected using an inverted microscope (AMG EVOS, USA). To recognize the P compartment boundary before statistical analysis of the invasive cell area, Ci was stained as the A compartment marker (Fig. S1). The invasive cell area in the P compartment of wing discs was calculated by the ImageJ program (National Institutes of Health). Statistical figures were generated by the GraphPad Prism 5 project.

### TUNEL assay

The wing discs were dissected from wandering third-instar larvae in PBS. The discs were fixed in 4% paraformaldehyde for 20 min and washed with PBST (0.2% Triton100) three times for 45 min at room temperature. TUNEL (TdT-mediated dUTP Nick-End Labeling) staining was performed using the *in situ* Cell Death Detection Kit (TMR red) produced by Sigma-Aldrich (Cat No. 12156792910).

## Supplementary Material

Supplementary information
