# Supplementary material for: The transcription factor Spalt and human homologue SALL4 induce cell invasion via the dMyc-JNK pathway in Drosophila
Source: Biol Open. 2020 Mar 24;9(3):bio048850. doi: 10.1242/bio.048850 (PMC7104861; doi:10.1242/bio.048850)
Supplement: Supplementary information [file biolopen-9-048850-s1.pdf]

Supplementary Information

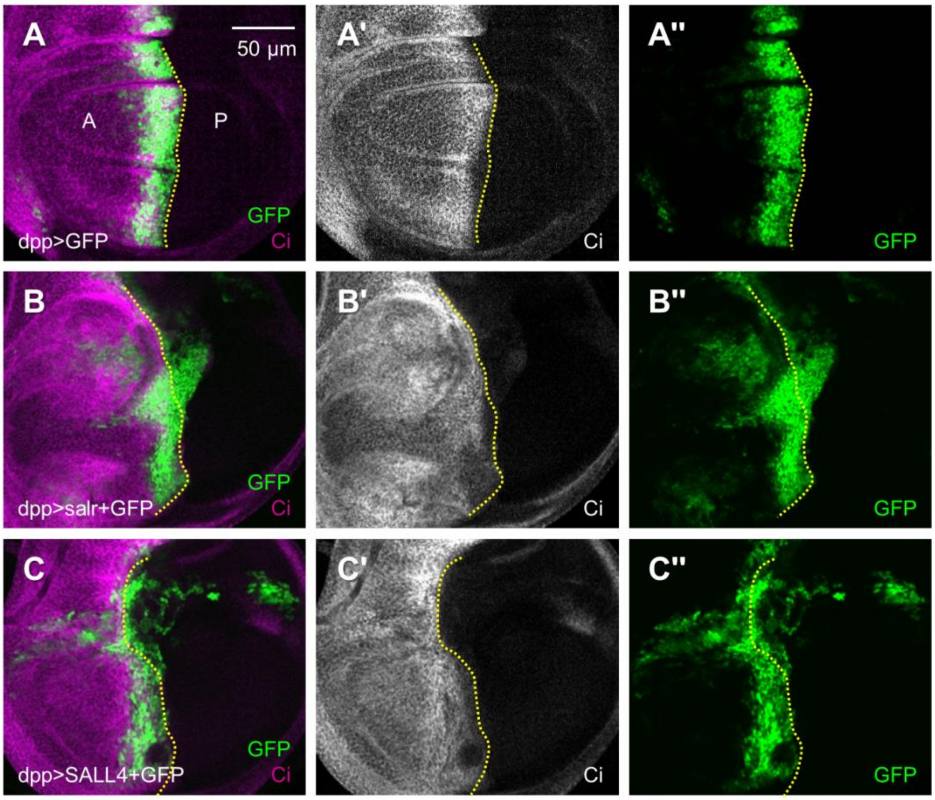

Fig. S1. Anterior-expressed GFP cells invade into the posterior compartment.

(A) The *dpp-Gal4*-driven GFP was expressed in the anterior compartment. Cubitus interruptus (Ci) represents the anterior cell fate. Dashed lines indicate the boundary between A and P compartment. (B, C) When *salr/SALL4* was ectopically expressed in the *dpp-Gal4* domain, GFP-expressing cells were present in the posterior compartment where Ci was absent. Scale bar: 50 µm.

Biology Open • Supplementary information

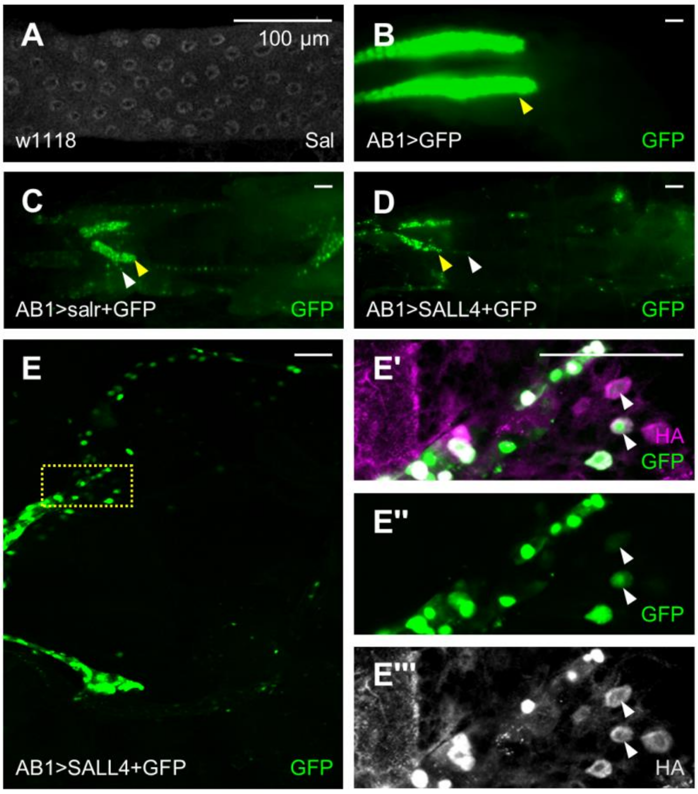

Fig. S2. ***sal/SALL4*** **induces cell movement in the salivary gland.**

(A) The endogenous localization of Sal in the salivary gland. (B) Cells expressing GFP in the *AB1-Gal4* domain did not migrate out of their expression domain. (C, D) Cells expressing *salr* or *SALL4* invaded into other tissues. (E) Cells overexpressing *SALL4* were migrated into the body in the dissected larvae. The anti-HA antibody was well co-localized with the GFP marker (E'). The yellow arrowheads indicate the salivary gland and the white arrowheads indicate the invading cells. Scale bars: 100 µm.

Biology Open • Supplementary information

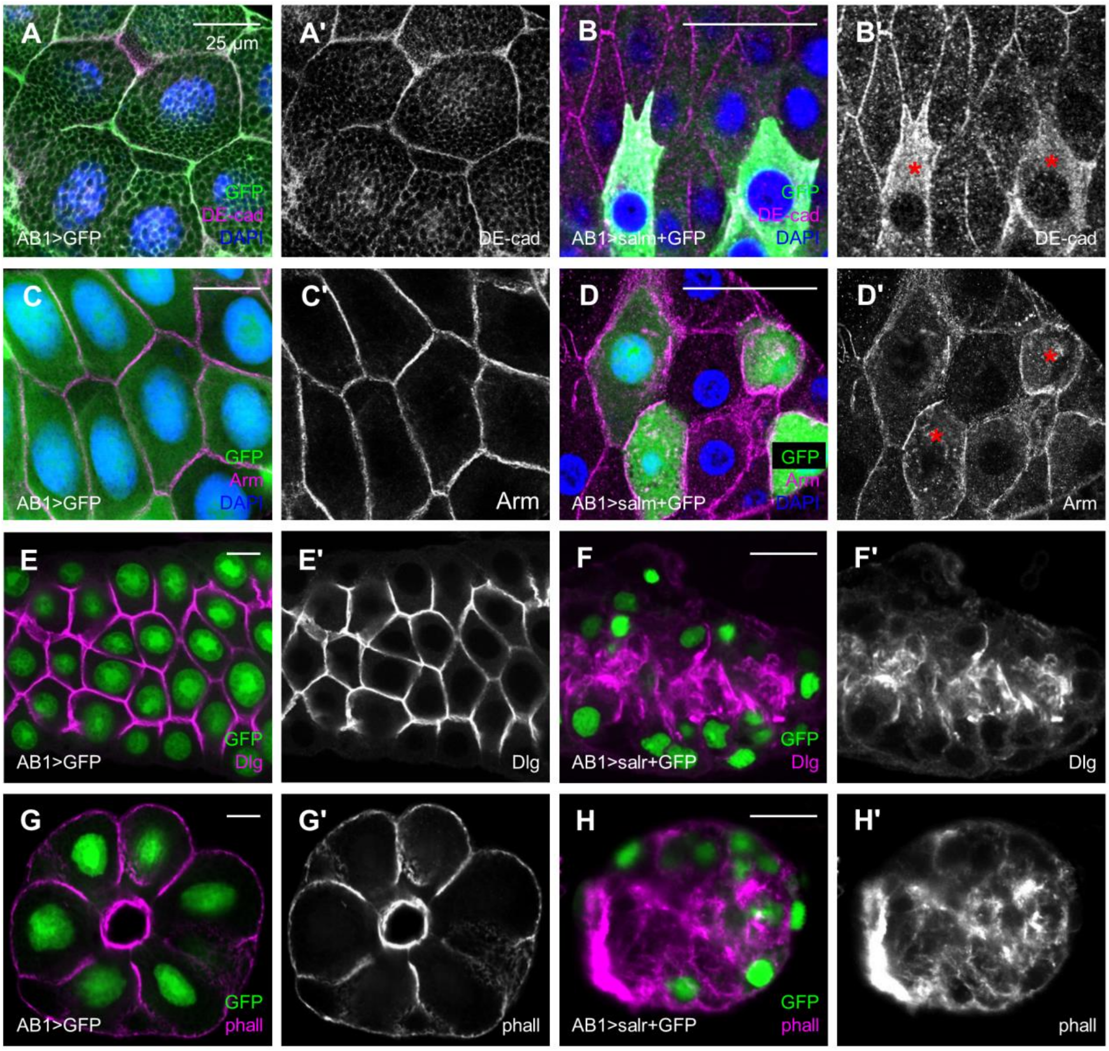

Fig. S3. Cell adhesion molecules and polarity proteins are regulated by *sal/SALL4* in the salivary gland.

(A) DE-cad was localized to the apical cell membrane in the salivary gland. (B) DE-cad was re-localized to the cytoplasm in *salm*-expressing cells. (C, D) Arm was re-distributed in *salm*-expressing cells. The red stars indicate the increased expression of DE-cad or Arm in cytoplasm. (E) In the wild-type salivary gland, Dlg was localized to the apical side. (F) The polar distribution of Dlg was lost when overexpressing *salr*. (G, H) In the cross-section of the salivary gland, F-actin localization was disordered when overexpressing *salr*. Scale bars: 25 μm.

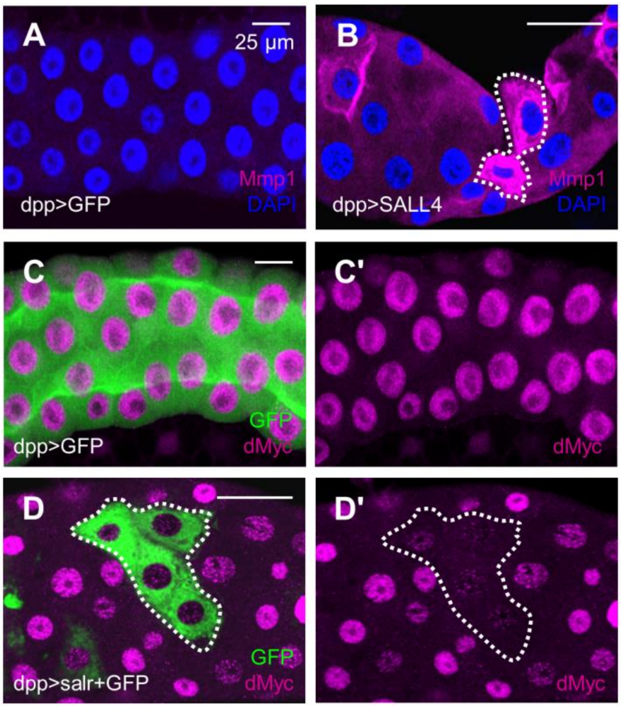

Fig. S4. The JNK signaling is activated, while dMyc is repressed by *sal/SALL4* in the salivary gland.

(A) Low Mmp1 expression in the control salivary gland. (B) Overexpression of *SALL4* promoted Mmp1 expression. (C, D) dMyc was inhibited in cells overexpressing *salr*. The dashed circles indicate the *dpp-Gal4* expressing cells where *salr*/*SALL4* was overexpressed. GFP is unevenly expressed in the salivary glands when *sal* was overexpressed, which is caused by an unknown mechanism. Scale bars: 25 µm.

Biology Open • Supplementary information

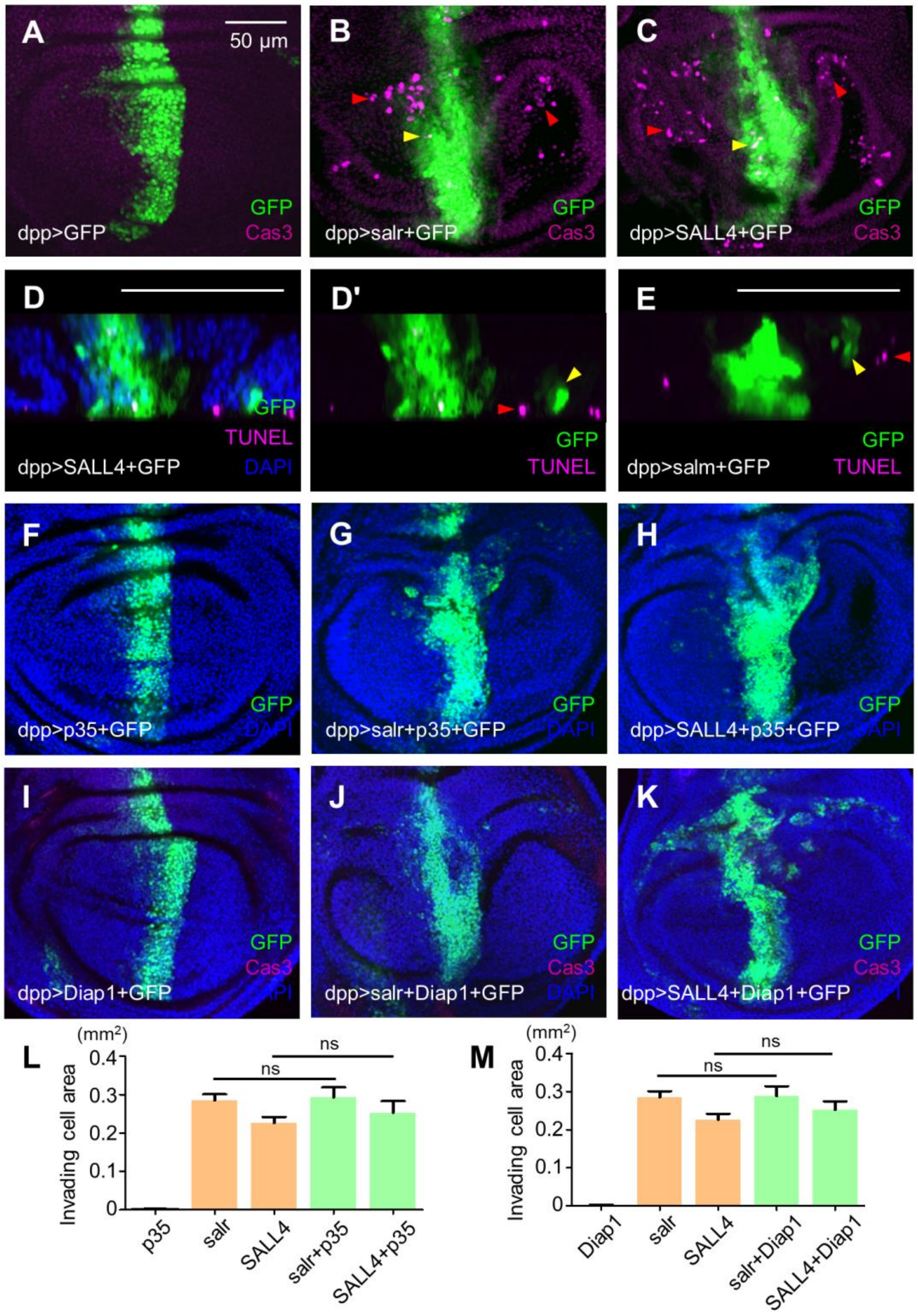

Fig. S5. ***sal/SALL4*-induced cell invasion is independent of cell death.**

(A) Wild-type wing disc cells had no obvious apoptosis as indicated by Cas3 staining. (B, C) Apoptotic cells were significantly increased in the wing disc overexpressing *salr* or *SALL4*. The yellow arrowheads mark the Cas3 activation in *sal*/*SALL4*-overexpessing cells, while red arrowheads mark the non-autonomously-expressed Cas3. (D, E) TUNEL analysis showed that the dead cells were not colocalized with the migrating cells in the x-z scans. The yellow arrowheads show the migrating cells and red arrowheads show the non-autonomous labeling by TUNEL. (F–H) Overexpression of *p35* did not rescue cell invasion induced by *salr* or

Biology Open • Supplementary information

SALL4. (I–K) Cell invasion induced by *salr* or *SALL4* was not significantly reduced by overexpressing *Diap1*. (L, M) Quantification of the invading cell area. Each genotype was quantified for 30 wing discs. n.s. means no significance (pairwise comparison of t-tests). Error bars indicate s.e.m. Scale bars: 50 μm.

Biology Open • Supplementary information
